# W-Modified TiAlN Coatings with Compact Non-Columnar Structure for Enhanced Hydrogen Barrier Protection of NdFeB Magnets

**DOI:** 10.3390/ma19153265

**Published:** 2026-08-02

**Authors:** Wanliang Zhang, Kaiyu Zhang, Chengshuang Zhou, Lin Zhang

**Affiliations:** Institute of Material Forming and Control Engineering, Zhejiang University of Technology, Hangzhou 310014, China; zhwl0525@gmail.com (W.Z.); 2111825057@zjut.edu.cn (K.Z.); zhoucs@zjut.edu.cn (C.Z.)

**Keywords:** TiAlN-W coating, hydrogen permeation barrier, W incorporation, electrochemical hydrogen permeation, NdFeB magnet

## Abstract

W-modified TiAlN coatings were developed as hydrogen permeation barrier coatings for magnetic materials. TiAlN and TiAlN-W coatings with comparable thicknesses of approximately 1.5 μm were deposited on Fe and NdFeB substrates by physical vapor deposition. GIXRD and SEM results showed that W modification was associated with a change from weakly crystalline, columnar TiAlN to a more compact, low-crystallinity W-modified Ti–Al–N coating with amorphous/nanocrystalline features inferred from the broad diffraction response. XPS analysis identified low-valence W-related species, such as W–N/W^n+^-related bonding, together with overlapping Ti 3p/W–O_x_ contributions and surface oxide/oxynitride species, indicating a modified surface and near-surface chemical environment. Electrochemical hydrogen permeation tests on Fe substrates showed that TiAlN reduced the steady-state current density from 2.54 ± 0.86 to 0.55 ± 0.08 μA cm^−2^, corresponding to a permeation reduction factor of 4.68 ± 0.16. In contrast, TiAlN-W showed no obvious hydrogen breakthrough during the 15,000 s test period, and the current density remained below the practical detection limit of 0.01 μA cm^−2^, giving a lower-bound permeation reduction factor of >250. In high-pressure H_2_ exposure tests, the TiAlN-W-coated NdFeB sample remained macroscopically intact after exposure to 5 MPa H_2_ at 23 °C for 24 h, whereas the uncoated and TiAlN-coated samples were pulverized. The markedly improved hydrogen permeation resistance is associated primarily with the more compact cross-sectional morphology and W-modified coating structure.

## 1. Introduction

Nd–Fe–B permanent magnets are key functional materials for high-power-density electrical machines because of their high-energy product. They are widely used in electric vehicle drive motors, wind-power generators and compact electromechanical systems [1,2,3]. With the rapid development of hydrogen technologies, permanent-magnet components may also be used in hydrogen-related equipment, such as fuel-cell air compressors and hydrogen recirculation pumps [4,5]. In these applications, magnetic materials may operate near hydrogen-containing media or under conditions where hydrogen ingress cannot be ignored. Therefore, the hydrogen-related reliability of magnetic materials has become an important issue for advanced energy systems.

Sintered NdFeB is sensitive to hydrogen. Hydrogen can react with Nd-rich grain-boundary phases and may further affect the Nd_2_Fe_14_B matrix. This process can induce hydride formation, grain-boundary cracking, interfacial debonding and pulverization [6,7,8]. These degradation processes can damage the structural integrity of the magnet and reduce its functional stability. Therefore, suppressing hydrogen ingress is necessary for NdFeB components used in hydrogen-related service environments.

Surface coatings are widely used to protect NdFeB magnets. Existing studies mainly focus on corrosion protection. Typical coating systems include metallic, polymeric, ceramic and composite coatings, and their performance is often evaluated by electrochemical corrosion tests or salt-spray exposure [9,10,11,12,13]. These coatings are usually designed to block corrosive species such as O_2_, H_2_O and Cl^−^. In contrast, coatings designed specifically for hydrogen permeation resistance on NdFeB magnets are still limited [8]. The relationship among coating composition, microstructure, defect structure, hydrogen transport and magnet integrity remains insufficiently understood. This gap calls for hydrogen-barrier coating strategies suitable for NdFeB magnets.

Hydrogen-barrier coatings have been studied in different material systems, including metallic coatings, ceramic coatings, polymer-based coatings and hybrid or multilayer coatings [14,15]. Their barrier performance depends not only on intrinsic hydrogen solubility and diffusivity, but also on defects, interfaces and diffusion-path continuity. Pinholes, cracks and connected grain boundaries can act as fast hydrogen transport channels. Dense ceramic coatings are attractive because they can provide high diffusion resistance and good chemical stability. However, their effectiveness is strongly affected by microstructural defects and coating/substrate compatibility [16,17,18,19]. Therefore, controlling the coating growth structure is critical for developing efficient hydrogen permeation barriers.

TiAlN is a promising ceramic hard coating for this purpose. It is a mature PVD nitride coating with high hardness, good thermal stability and oxidation resistance [20]. Recent computational and experimental studies also suggest that TiAlN has potential resistance to hydrogen uptake and transport [16,20,21]. However, monolithic PVD TiAlN coatings may contain deposition-induced defects. In particular, columnar boundaries can form continuous through-thickness pathways for hydrogen migration. Thus, the key challenge is to obtain a compact and non-columnar TiAlN-based coating rather than relying only on the intrinsic properties of TiAlN.

W modification offers a possible route to tailor the growth structure of TiAlN. Previous studies on W-modified Ti–Al–N-based coatings have shown that W can affect coating growth, defect formation, phase stability and thermo-mechanical or wear performance [22,23]. These studies demonstrate that W is an effective alloying element for modifying Ti–Al–N coating structure and properties. However, most previous works focused on hard protective coatings for mechanical, tribological or thermal applications. The role of W-modified TiAlN coatings as hydrogen permeation barriers has not been sufficiently clarified, especially for hydrogen-sensitive NdFeB magnets. Tungsten has low hydrogen solubility and low bulk hydrogen transport, but hydrogen migration may be accelerated along grain boundaries in W-modified materials [24,25]. This indicates that the final hydrogen barrier performance depends strongly on the coating microstructure. Therefore, introducing W into TiAlN may help suppress continuous columnar diffusion channels and modify the local bonding environment of the coating.

In this work, TiAlN and W-modified TiAlN coatings with comparable thicknesses were prepared by PVD. Fe substrates were used for quantitative electrochemical hydrogen permeation measurements, while NdFeB substrates were used for high-pressure hydrogen exposure tests to assess application relevance. The effects of W modification on phase structure, morphology, elemental composition, surface chemical states and hydrogen permeation behavior were investigated. The novelty of this work lies in the direct comparison of TiAlN and W-modified TiAlN coatings with comparable thicknesses under identical hydrogen permeation conditions, and in the application-oriented validation of W-modified TiAlN as a hydrogen permeation barrier coating for NdFeB magnets. The aim is to clarify whether the W-induced compact coating structure and modified surface chemistry can effectively suppress hydrogen permeation and improve the structural integrity of NdFeB magnets in hydrogen-containing environments.

## 2. Materials and Methods

### 2.1. Preparation of Substrates and Coatings

Commercially pure Fe substrates (>99.9%) were cut into plates with dimensions of 30 × 30 × 2 mm^3^ by wire electrical discharge machining. Commercial NdFeB magnets with the same dimensions of 30 × 30 × 2 mm^3^ were used as application substrates. Before coating deposition, the NdFeB magnets were demagnetized in a vacuum tube furnace at 180 °C for 20 min. All substrates were mechanically ground using SiC abrasive papers with grit sizes of 1000, 1500 and 2000. They were then ultrasonically cleaned in ethanol and acetone (GoldWallReagent, Kunshan City, China) baths for 20 min. After cleaning, the substrates were dried using an air blower.

Single-side polished Si wafers were used for GIXRD analysis to reduce substrate-related diffraction interference. This is particularly important because the coating thickness was only approximately 1.5 ± 0.2 μm, and Fe or NdFeB substrates can produce strong diffraction signals that may obscure the weak diffraction response of the thin coatings.

TiAlN and TiAlN-W coatings were deposited using a PVD JGP-450 deposition system (Shenyang Sky Technology Development Co., Ltd., Shenyang City, China). The coatings were prepared by co-sputtering from separate elemental targets rather than alloyed targets. For TiAlN, separate Ti and Al targets were used. For TiAlN-W, an additional W target was introduced together with the Ti and Al targets. The same Ar/N_2_ flow ratio was used for both coatings to maintain a consistent reactive sputtering atmosphere and to isolate the effect of W modification.

The coating thickness was controlled at approximately 1.5 μm for all samples. The TiAlN-W deposition condition was first fixed, and the TiAlN deposition time was then extended to obtain a comparable coating thickness. This adjustment was necessary because TiAlN showed a lower deposition rate than TiAlN-W under the present conditions, mainly due to the additional sputtering flux from the W target during TiAlN-W deposition. Based on the measured final thicknesses, the average deposition rates were estimated to be approximately 8.3 nm min^−1^ for TiAlN and 37.5 nm min^−1^ for TiAlN-W. The final coating thicknesses were confirmed by cross-sectional SEM measurements at more than five positions for each coating, and both coatings were within approximately 1.5 ± 0.2 μm. Therefore, the hydrogen permeation comparison was performed using coatings with comparable thicknesses rather than relying on deposition time alone.

For the Fe substrates used in electrochemical hydrogen permeation tests, the coatings were deposited on one side only. For the NdFeB substrates used in hydrogen exposure tests, the coatings were deposited to fully cover the sample surface. The detailed deposition parameters are listed in Table 1.

### 2.2. Characterization Methods

The phase structures of the TiAlN and TiAlN-W coatings were analyzed by grazing-incidence X-ray diffraction (GIXRD, SmartLab 9 kW, Rigaku Corporation, Akishima, Tokyo, Japan). The grazing incidence angle was fixed at 1°.

The surface and cross-sectional morphologies of the coatings were observed using a field-emission scanning electron microscope (FESEM, ZEISS ΣIGMA, Carl Zeiss Microscopy GmbH, Jena, Germany). The elemental compositions of the coatings were analyzed by energy-dispersive X-ray spectroscopy (EDS).

The chemical states of the elements in the TiAlN and TiAlN-W coatings were examined by X-ray photoelectron spectroscopy (XPS, Thermo Scientific ESCALAB 250Xi, East Grinstead, UKU) with a monochromatic Al K_α_ X-ray source. Before data acquisition, the samples were etched by Ar ions for 50 s. The Ar^+^ etching rate was approximately 10 nm min^−1^, corresponding to an estimated etched depth of approximately 8 nm. Since the etching rate may vary with coating composition, this value is used only as an approximate depth estimate. Therefore, the XPS results are discussed as surface/near-surface chemical information rather than depth-resolved chemical profiles. The pass energy for the core-level spectra was set to 30 eV. The step size and number of scans were 0.05 eV and 5, respectively.

The XPS spectra were fitted using XPSPEAK 4.1 software. A Gaussian–Lorentzian mixed line shape and a Shirley background were used for peak fitting and peak position determination.

### 2.3. Electrochemical Hydrogen Permeation Measurements

Electrochemical hydrogen permeation measurements were carried out using a classical Devanathan–Stachurski double-cell system. A 0.2 mol L^−1^ NaOH solution was used as the electrolyte in both the hydrogen charging cell and the hydrogen detection cell. Disc-shaped Fe-based specimens with a diameter of 30 mm were used for the permeation tests.

Each test was repeated three times to ensure reproducibility. To ensure reliable and reproducible electrochemical hydrogen permeation measurements, all Fe-based specimens were electroplated with Ni layers on the hydrogen charging side. The Ni plating solution contained 125 g L^−1^ NiSO_4_·7H_2_O, 22.5 g L^−1^ NiCl_2_·6H_2_O and 20 g L^−1^ H_3_BO_3_, all of which were purchased from Shanghai Wokai Biotechnology Co., Ltd. (Shanghai, China). The electroplating was performed at a current density of 10 mA cm^−2^ for 3 min, producing a Ni layer with a thickness of approximately 0.5 μm. The same Ni electroplating process was applied to all Fe-based specimens to provide identical hydrogen charging conditions. Because the Ni layer was much thinner than the Fe substrate and was located on the hydrogen charging side rather than the detection side, it was not expected to act as the rate-limiting barrier in the present Devanathan–Stachurski system. After one-side polishing and Ni electroplating, the thicknesses of the three Fe-based specimens were 1.61, 1.63 and 1.62 mm, respectively. Before assembly, the non-Ni-plated side of each specimen was cleaned by pickling in 10% HCl solution (Shanghai Wokai Biotechnology Co., Ltd. Shanghai, China) and then rinsed with ethanol–acetone solution. The specimens were dried before being mounted in the permeation cell.

To minimize edge effects, only a circular area with a diameter of 20 mm was exposed to the electrolyte. The specimen served as the working electrode. In the hydrogen detection cell, a saturated calomel electrode and a Pt sheet were used as the reference electrode and counter electrode, respectively. Before each test, the background current density in the hydrogen detection cell was stabilized. The stabilized background/noise level of the present electrochemical permeation system was approximately 0.008–0.01 μA cm^−2^. Therefore, 0.01 μA cm^−2^ was used as a conservative practical lower detection limit for the permeation current density in this work. Hydrogen charging was then performed at a constant current density of 5 mA cm^−2^. All permeation tests were conducted at room temperature, which was maintained at 23 °C.

The hydrogen permeation current density was recorded as a function of time. The hydrogen permeation reduction factor (PRF) was used to evaluate the hydrogen permeation barrier efficiency of the coatings. According to the commonly used definition for permeation barriers, the PRF was calculated from the ratio of the steady-state permeation current density of the uncoated substrate to that of the coated specimen:

(1) $$PRF=\frac{i_{sub}}{i_{coat}}$$

where $i_{sub}$ is the steady-state hydrogen permeation current density of the bare Fe substrate, and $i_{coat}$ is the steady-state hydrogen permeation current density of the coated Fe specimen. All PRF values were calculated under the same testing conditions. When the permeation current of a coated specimen remained at the background level, the steady-state current density was reported as lower than 0.01 μA cm^−2^, and the PRF was reported as a lower-bound value rather than an exact value.

For measurable permeation currents, the uncertainty of PRF was estimated by error propagation:

(2) $$\sigma_{PRF}=PRF\sqrt{{\left(\frac{\sigma_{sub}}{i_{sub}}\right)}^{2}+{\left(\frac{\sigma_{coat}}{i_{coat}}\right)}^{2}}$$

where $\sigma_{sub}$ and $\sigma_{coat}$ are the standard deviations of the steady-state permeation current densities of the bare Fe substrate and coated specimen, respectively. For TiAlN-W, conventional error propagation was not applied because the permeation current was below the practical detection limit. Therefore, its PRF was conservatively reported as a lower-bound value.

### 2.4. High-Pressure Hydrogen Exposure of NdFeB Magnets

**Figure 1:**
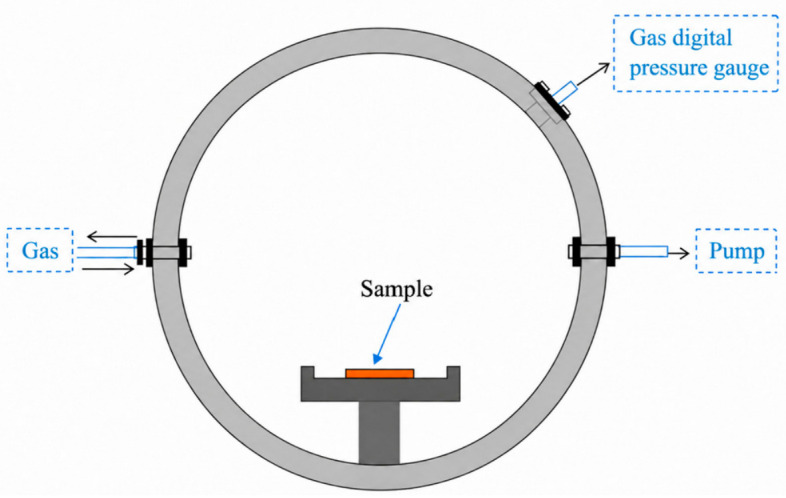
Schematic diagram of the high-pressure hydrogen exposure chamber used for NdFeB magnet testing. The chamber was equipped with gas inlet/outlet ports, a digital gas pressure gauge and a vacuum port connected to a mechanical vacuum pump.

High-pressure hydrogen exposure tests were carried out to evaluate the application potential of the coatings on NdFeB magnets. The chamber was equipped with gas inlet/outlet ports, a digital pressure gauge and a vacuum port. During exposure, the internal pressure was monitored using the digital pressure gauge. After exposure, hydrogen was released through the outlet according to the laboratory safety procedure. The schematic in Figure 1 is simplified and is intended to show the main testing configuration rather than all engineering safety components.

Before hydrogen exposure, the chamber was cleaned by five cycles of nitrogen purging and evacuation. Each evacuation step was maintained for 20 min. This procedure was used to remove residual O_2_ and H_2_O from the chamber and to reduce the influence of gas impurities on the exposure test.

Uncoated NdFeB, TiAlN-coated NdFeB and TiAlN-W-coated NdFeB samples were placed in the chamber. The coated samples were fully covered by the corresponding coatings before exposure. After the purging and evacuation process, high-purity H_2_ was introduced into the chamber until the pressure reached 5 MPa. The samples were exposed to 5 MPa H_2_ at 23 °C for 24 h. After exposure, the samples were removed from the chamber and their macroscopic appearance was recorded under the same lighting and environmental conditions.

This test was used as an application-oriented qualitative assessment of hydrogen-induced pulverization resistance. It was not used to quantify hydrogen uptake or hydrogen permeability in NdFeB.

## 3. Results and Discussion

### 3.1. Phase Structure of the Coatings

**Figure 2:**
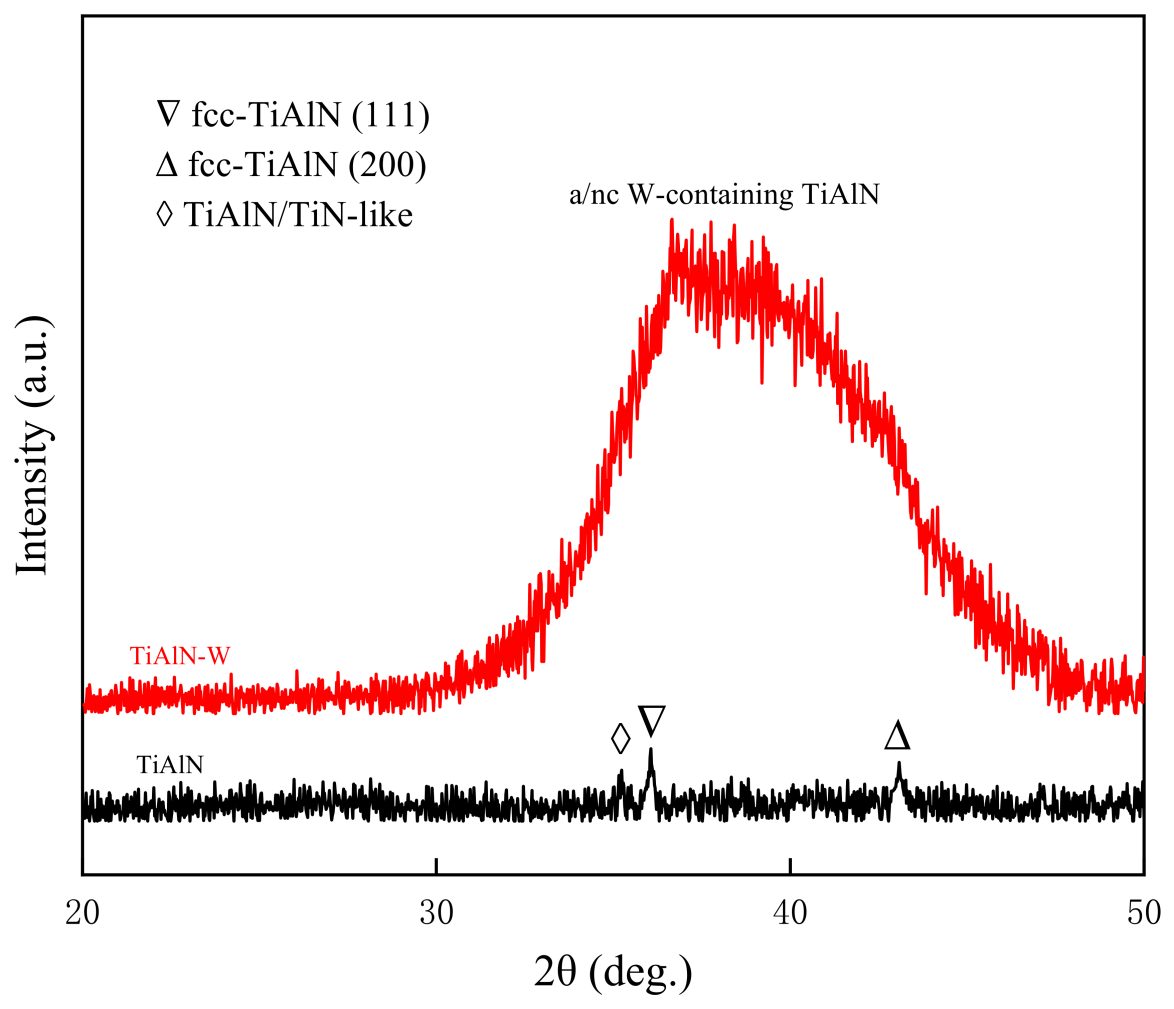
GIXRD patterns of TiAlN and TiAlN-W coatings deposited on Si (100) substrates at a grazing incidence angle of 1°. The patterns are vertically shifted for clarity. The TiAlN coating shows weak diffraction peaks corresponding to fcc-TiAlN/TiN-like phases. In contrast, the TiAlN-W coating exhibits a broad diffraction band, suggesting an amorphous/nanocrystalline W- modified TiAlN structure. Here, a/nc denotes amorphous/nanocrystalline.

Figure 2 shows the GIXRD patterns of the TiAlN and TiAlN-W coatings measured at a grazing incidence angle of 1°. The TiAlN coating exhibits weak diffraction peaks that can be assigned to fcc-TiAlN/TiN-like phases. The reflections related to fcc-TiAlN (111) and (200) are observed, but their low intensity indicates limited crystallinity of the TiAlN coating.

After W incorporation, the diffraction pattern changes significantly. The TiAlN-W coating does not show sharp crystalline peaks. Instead, a broad diffraction band is observed over the corresponding 2θ range. This feature suggests that W incorporation reduces the crystallinity of the TiAlN coating and leads to a low-crystallinity W-modified Ti–Al–N structure with amorphous/nanocrystalline features. Since HRTEM was not available in the present revision, the crystallite size distribution could not be directly determined. Therefore, the amorphous/nanocrystalline nature is discussed based on the broad GIXRD response rather than direct lattice imaging. The broadening may be related to local lattice distortion and interrupted crystal growth caused by W addition. This structural change is consistent with the SEM results, where the TiAlN-W coating shows a more compact cross-sectional morphology than the TiAlN coating.

It should be noted that the GIXRD measurements were performed on coatings deposited on Si substrates to reduce diffraction interference from Fe or NdFeB substrates. Substrate materials may affect film nucleation, texture, residual stress and interfacial structure [26]. Therefore, the present GIXRD results are mainly used to compare the relative structural difference between TiAlN and TiAlN-W deposited under the same process conditions. Direct confirmation of the coating structure and interface continuity on Fe or NdFeB substrates would require further substrate-specific GIXRD or TEM characterization.

### 3.2. Morphology and Elemental Composition

**Figure 3:**
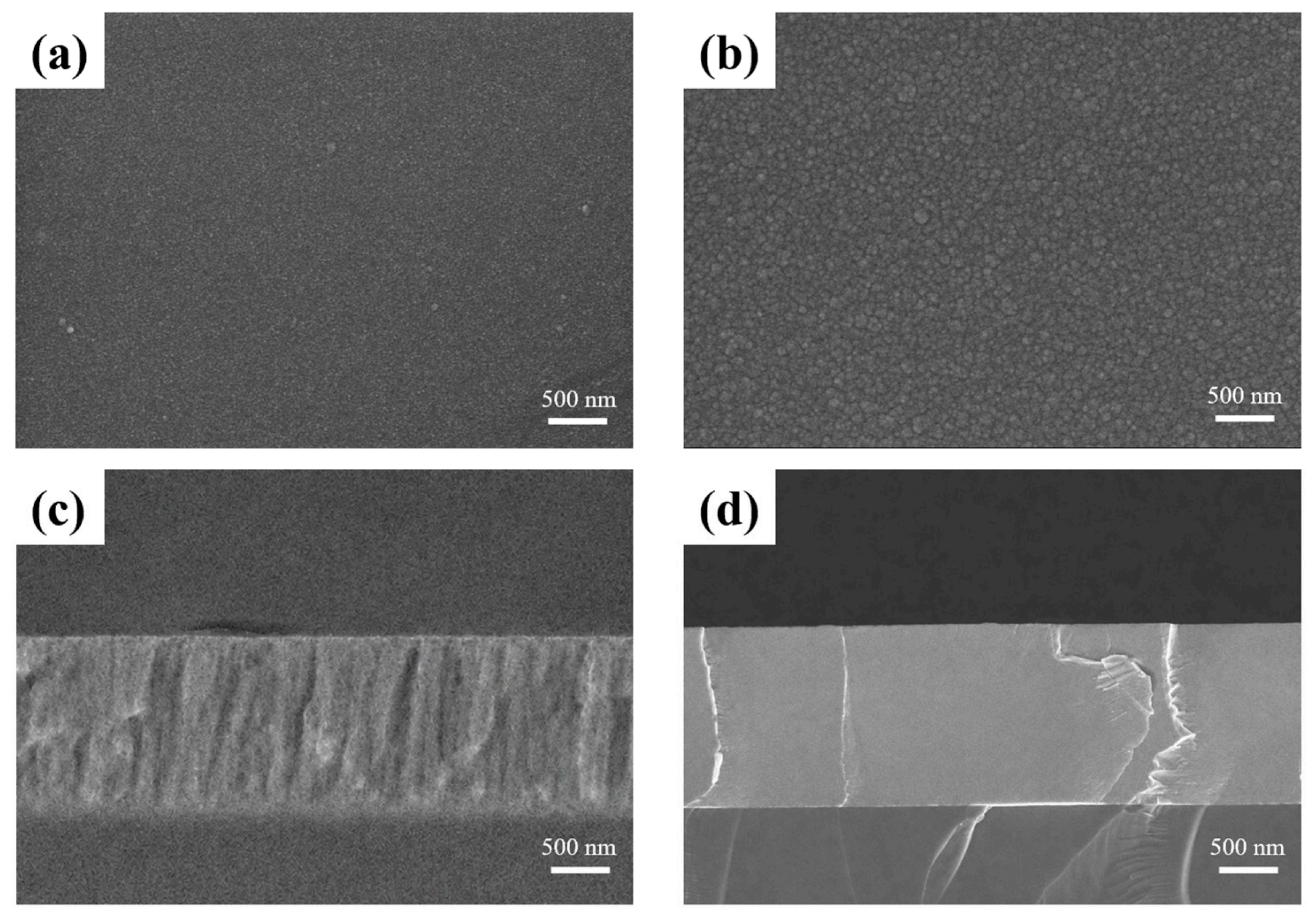
Surface and cross-sectional SEM images of TiAlN and TiAlN-W coatings with comparable thicknesses of approximately 1.5 μm: (**a**,**c**) TiAlN and (**b**,**d**) TiAlN-W. Panels (**a**,**b**) show the surface morphologies, while panels (**c**,**d**) show the cross-sectional morphologies. The corresponding EDS compositions are listed in Table 2.

Figure 3 shows the surface and cross-sectional SEM images of the TiAlN and TiAlN-W coatings. Both coatings are continuous and have comparable thicknesses of approximately 1.5 μm. Cross-sectional SEM measurements were performed at more than five different locations for each coating. The measured thicknesses of both TiAlN and TiAlN-W coatings were within 1.5±0.2 μm, indicating limited thickness variation and good thickness comparability between the two coatings. This thickness similarity is important for the following hydrogen permeation comparison, because it reduces the influence of coating thickness on the barrier performance.

The TiAlN coating shows a relatively smooth surface with fine granular features (Figure 3a). No obvious surface cracks or large defects are observed. However, its cross-section displays a clear columnar morphology (Figure 3c). The column boundaries extend along the film growth direction. This morphology is typical for many PVD nitride coatings. Such columnar boundaries may act as preferential transport paths for small atoms such as hydrogen.

After W incorporation, the surface morphology changes clearly. The TiAlN-W coating shows a coarser granular surface than the TiAlN coating (Figure 3b). These surface features should be regarded as mound-like or granular surface structures. They do not directly indicate a porous coating. This is supported by the cross-sectional SEM image. As shown in Figure 3d, the TiAlN-W coating exhibits a much more compact cross-section. The columnar structure is strongly suppressed, and no obvious open columnar voids are observed at the SEM resolution. The TiAlN-W coating shows a coarser granular surface than the TiAlN coating. Surface roughness may affect the real surface area, local wetting behavior and surface reaction sites during electrochemical testing [27]. However, a rougher surface does not necessarily indicate a more porous through-thickness structure. In the present case, the cross-sectional SEM image shows that TiAlN-W has a compact and less columnar morphology. Therefore, the coarse surface features are mainly regarded as surface granular or mound-like structures rather than evidence of open permeation paths through the coating.

The difference between the surface and cross-sectional morphologies suggests that W incorporation changes the coating growth mode. The TiAlN coating grows with a more evident columnar structure. In contrast, the TiAlN-W coating tends to form a denser and less columnar structure. This result is consistent with the GIXRD result, where TiAlN-W shows a broad diffraction band and reduced crystallinity. However, direct confirmation of nanoscale crystallite distribution and coating–substrate interface continuity would require future HRTEM analysis. The compact cross-sectional structure can decrease continuous through-thickness defects. It can also increase the tortuosity of hydrogen transport paths. The EDS results of Table 2 confirm the formation of a W-modified Ti–Al–N coating.

Overall, the SEM and EDS results show that W incorporation not only changes the elemental composition of TiAlN but also modifies its growth morphology. The TiAlN-W coating has a coarser surface but a denser cross-section. This structural feature is beneficial for suppressing hydrogen permeation, because it reduces continuous columnar boundaries and intercolumnar defects through the coating thickness.

### 3.3. Surface Chemical States

XPS was used to further examine the surface chemical states of the coatings. The fitting parameters are listed in Table S1.

**Figure 4:**
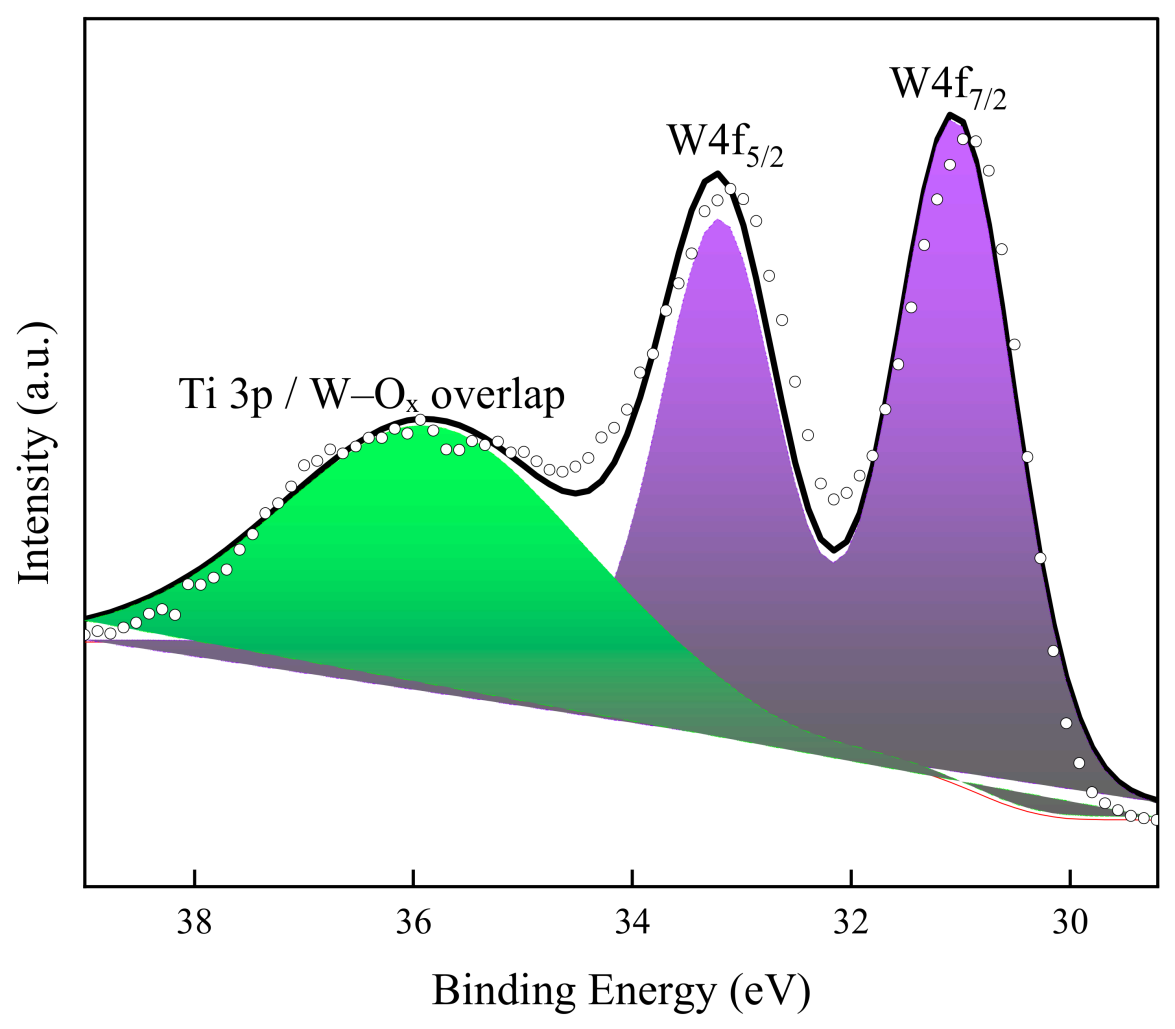
High-resolution W 4f XPS spectrum of the TiAlN–W coating. The open circles, black solid line, colored components, and background line represent the experimental data, fitted envelope, fitted components, and background, respectively. The W 4f doublet was fitted using a constrained spin–orbit doublet, with the W 4f_7/2_–W 4f_5/2_ separation fixed at approximately 2.15 eV and the W 4f_7/2_:W 4f_5/2_ area ratio constrained to 4:3. The low-binding-energy W 4f_7/2_ and high-binding-energy W 4f_5/2_ components are assigned to low-valence W-related species, such as W–N/W^n+^-related bonding. The broad component at higher binding energy is attributed to the overlap of Ti 3p and W–O_x_-related species. The binding energy was calibrated using the C 1s peak at 284.8 eV.

Figure 4 shows the high-resolution W 4f spectrum of the TiAlN-W coating. A clear W 4f spin–orbit doublet is observed after charge correction. During fitting, the W 4f doublet was constrained with a spin–orbit splitting of approximately 2.15 eV and an area ratio of W 4f_7/2_:W 4f_5/2_ = 4:3. These constraints were used to maintain the physical relationship between the W 4f_7/2_ and W 4f_5/2_ components and to avoid overfitting.

The lower-binding-energy component at approximately 31.0 eV is assigned to W 4f_7/2_, while the corresponding higher-binding-energy component is assigned to W 4f_5/2_. The position of the W 4f_7/2_ peak supports the presence of low-valence W-related species. Considering the N-containing deposition atmosphere and the metal–nitrogen contribution observed in the N 1s spectrum, this doublet can be associated with W–N/W^n+^-related bonding. However, the W 4f binding energy alone cannot fully distinguish W–N from all possible low-valence W states. Therefore, this component is described as low-valence W-related species, such as W–N/W^n+^-related bonding.

A broad component is also observed at higher binding energy. This feature is attributed to the overlap of Ti 3p and W–O_x_-related species. The Ti 3p contribution is significant because Ti is a major element in the coating and appears in the same binding-energy region as oxidized W-related components. Therefore, the high-binding-energy feature should not be interpreted as a single W oxide component. Instead, it indicates the coexistence of Ti 3p overlap and near-surface W–O_x_-related contributions. According to Table S1, the low-valence W-related doublet accounts for approximately 65.68% of the fitted W 4f/Ti 3p region, while the broad Ti 3p/W–O_x_-related contribution accounts for approximately 34.31%. Due to the Ti 3p/W–O_x_ overlap and possible sputter-induced reduction of W oxides during Ar^+^ etching, these values are discussed only as semi-quantitative trends rather than exact W chemical-state fractions. Future work may combine high-resolution XPS with reference compounds, angle-resolved or hard X-ray photoelectron spectroscopy, and synchrotron-based X-ray absorption spectroscopy such as XANES/EXAFS to better separate the Ti 3p/W–O_x_ overlap and clarify the local chemical coordination of W in TiAlN-W coatings.

**Figure 5:**
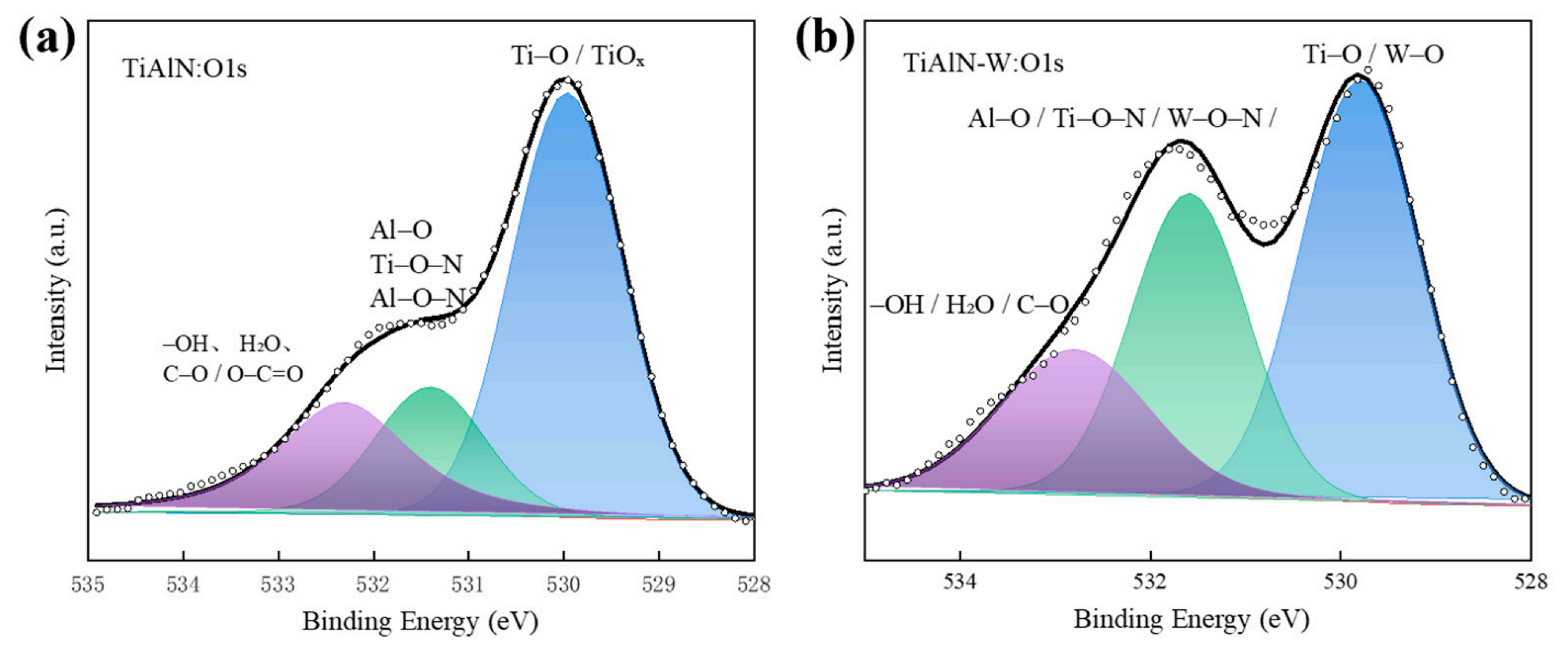
High-resolution O 1s XPS spectra of (**a**) TiAlN and (**b**) TiAlN-W coatings. The symbols and fitting components have the same meanings as those defined in Figure 4. The O 1s spectrum of TiAlN is fitted with components related to Ti–O/TiOx, Al–O, Ti–O–N, Al–O–N and adsorbed oxygen-containing species. The TiAlN-W coating shows additional oxygen bonding environments related to Ti–O/W–O and Al–O/Ti–O–N/W–O–N species.

Figure 5 compares the O 1s spectra of TiAlN and TiAlN-W. The TiAlN coating shows a dominant Ti–O/TiOx component. It also contains weaker components related to Al–O, Ti–O–N, Al–O–N and adsorbed oxygen-containing species. The O 1s spectrum of TiAlN-W is broader. It contains a Ti–O/W–O component and a pronounced intermediate component related to Al–O, Ti–O–N and W–O–N species. This indicates that W incorporation changes the surface oxygen bonding environment of TiAlN.

**Figure 6:**
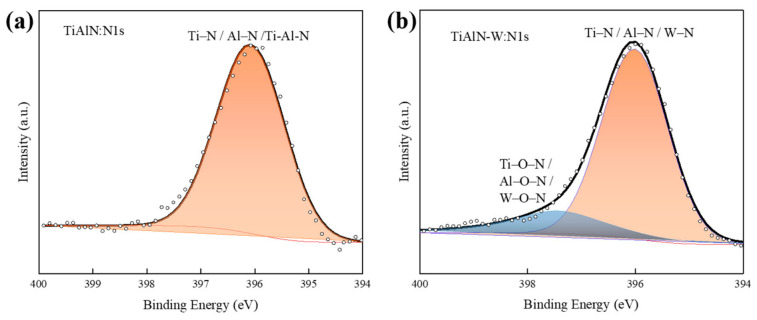
High-resolution N 1s XPS spectra of (**a**) TiAlN and (**b**) TiAlN-W coatings. The symbols and fitting components have the same meanings as those defined in Figure 4. The main N 1s peak of TiAlN is assigned to Ti–N, Al–N and Ti–Al–N bonding. For TiAlN-W, the main N 1s peak is assigned to Ti–N, Al–N and W–N bonding. The weak high-binding-energy component is related to Ti–O–N, Al–O–N or W–O–N species.

Figure 6 shows the N 1s spectra of the two coatings. The TiAlN coating presents a main N 1s peak assigned to Ti–N, Al–N and Ti–Al–N bonding. The TiAlN-W coating also shows a strong metal–nitrogen peak. This peak is assigned to Ti–N, Al–N and W–N bonding. A weak shoulder at higher binding energy is observed for TiAlN-W. It is related to Ti–O–N, Al–O–N or W–O–N species. The N 1s results confirm the formation of nitride bonding in both coatings. They also support the presence of W-related nitride or oxynitride species in TiAlN-W.

The relative areas listed in Table S1 provide semi-quantitative information on the surface chemical states. For W 4f, the low-valence W-related doublet accounts for approximately 65.68% of the fitted W 4f/Ti 3p region, while the Ti 3p/W–O_x_-related contribution accounts for approximately 34.31%. Due to the overlap between Ti 3p and W–O_x_ signals, these W-related fractions should be interpreted only semi-quantitatively. For O 1s, TiAlN is dominated by the Ti–O/TiO_x_ component, whereas TiAlN-W shows a larger contribution from oxide/oxynitride-related species. For N 1s, TiAlN-W contains a main Ti–N/Al–N/W–N component and a weak oxynitride-related shoulder, supporting the presence of W-related nitride and oxynitride surface species.

Overall, the XPS results show that W incorporation modifies the surface chemistry of TiAlN. The TiAlN-W coating contains low-valence W-related species, metal–nitrogen bonds and surface oxide/oxynitride species. These surface chemical states, together with the compact cross-sectional morphology, may contribute to the improved hydrogen barrier performance of the TiAlN-W coating.

### 3.4. Electrochemical Hydrogen Permeation Behavior

**Figure 7:**
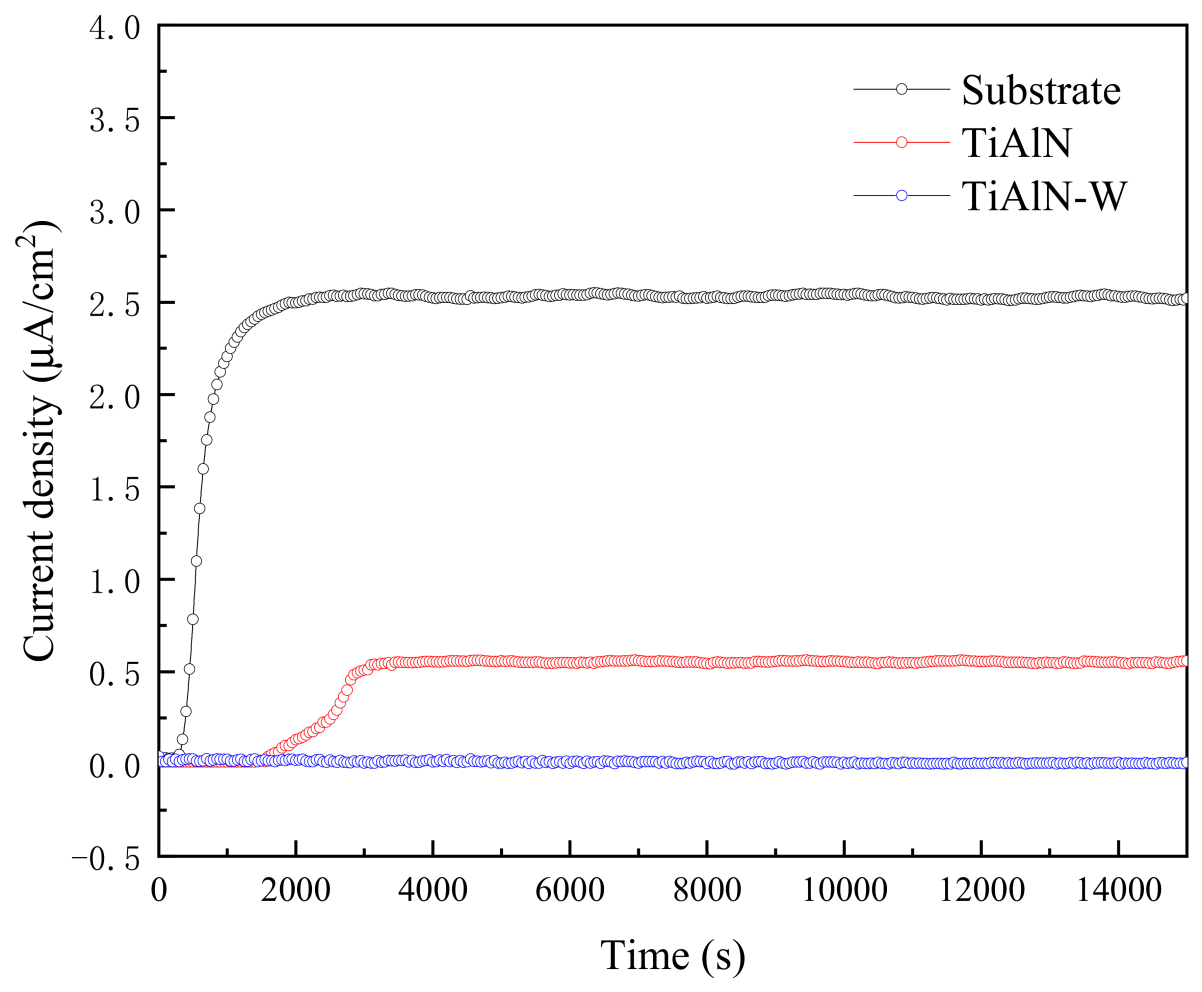
Representative electrochemical hydrogen permeation current density curves of the bare Fe substrate, TiAlN-coated Fe and TiAlN-W-coated Fe. The open symbols represent experimental data points, and the connecting solid lines are guides to the eye. Each curve was selected from three independent specimens. The quantitative values were calculated from the average of three measurements. The averaged steady-state current densities and permeation reduction factors are listed in Table 3.

Figure 7 shows representative electrochemical hydrogen permeation curves of the bare Fe substrate, TiAlN-coated Fe and TiAlN-W-coated Fe. The representative curves were selected from three independent specimens, and the averaged permeation parameters are summarized in Table 3. The full permeation curves of the three repeated tests are provided in Figure S1. The PRF was calculated from the ratio of the steady-state permeation current density of the bare Fe substrate to that of the coated sample as Equation (1).

The bare Fe substrate exhibited a rapid hydrogen permeation response. The breakthrough time was 270 ± 5 s, and the steady-state current density reached 2.54 ± 0.86 μA cm^−2^. This indicates fast hydrogen transport through the Fe substrate under the present testing condition.

The TiAlN coating reduced the permeation current and delayed hydrogen breakthrough. The steady-state current density decreased to 0.55 ± 0.08 μA cm^−2^, and the breakthrough time increased to 1431 ± 8s. The corresponding PRF was 4.68 ± 0.16. These results confirm that TiAlN can act as a hydrogen permeation barrier. However, a clear permeation signal was still detected, indicating that hydrogen transport was not fully suppressed.

The TiAlN-W coating showed the strongest barrier effect. No obvious hydrogen breakthrough was detected during the test period of 15,000 s. The current density remained close to the stabilized background/noise level of the permeation cell, which was approximately 0.008–0.01 μA cm^−2^. Therefore, 0.01 μA cm^−2^ was used as a conservative practical lower detection limit, and the steady-state current density of TiAlN-W was reported as <0.01 μA cm^−2^. The breakthrough time could not be determined within the test duration. As shown in Figure S1, the three repeated TiAlN-W curves remained below the detection limit, further confirming the reproducibility of the near-background current response.

Since the TiAlN-W current was close to the detection limit, its PRF was reported as a lower-bound value. Using $0.01\;\mu Acm^{-2}$ as the conservative detection limit and $2.54\;\mu Acm^{-2}$ as the mean steady-state current density of the bare Fe substrate, the lower-bound PRF was calculated to be >254. Therefore, the PRF of TiAlN-W was conservatively reported as >250. The exact PRF may be higher, but it cannot be reliably quantified due to the sensitivity limitation of the electrochemical permeation technique. Since the two coatings had comparable thicknesses of 1.5±0.2 μm, the large difference in permeation behavior cannot be mainly attributed to a coating thickness effect.

To further clarify the significance of the present result, the PRF of TiAlN-W was compared with representative hydrogen barrier coatings reported in the literature, as summarized in Table 4. It should be noted that PRF values are affected by coating thickness, substrate material, test temperature, hydrogen source, testing method and defect density. Therefore, this comparison should be regarded as semi-quantitative rather than an absolute ranking. A charging current density higher than 5 mA cm^−2^ was not examined in the present study to maintain identical testing conditions among all specimens; higher driving forces and longer test durations will be considered in future work to determine the ultimate breakthrough behavior of TiAlN-W.

As shown in Table 4, conventional PVD TiN coatings generally show limited PRF values at room temperature, while dense Al-containing nitride coatings, multilayer nitride coatings and amorphous ceramic coatings can exhibit much higher barrier performance under specific testing conditions. Compared with these reported systems, the present TiAlN-W coating achieves a lower-bound PRF of >250 at 23 °C with a thickness of only 1.5±0.2 μm. More importantly, W incorporation increases the PRF of TiAlN from 4.68±0.16 to >250 under identical testing conditions. This comparison highlights that W incorporation is an effective strategy for improving TiAlN-based hydrogen barrier coatings by suppressing continuous columnar diffusion channels and promoting a compact W-modified Ti–Al–N structure, as further discussed in Section 3.6.

### 3.5. Application Validation on NdFeB Magnets

**Figure 8:**
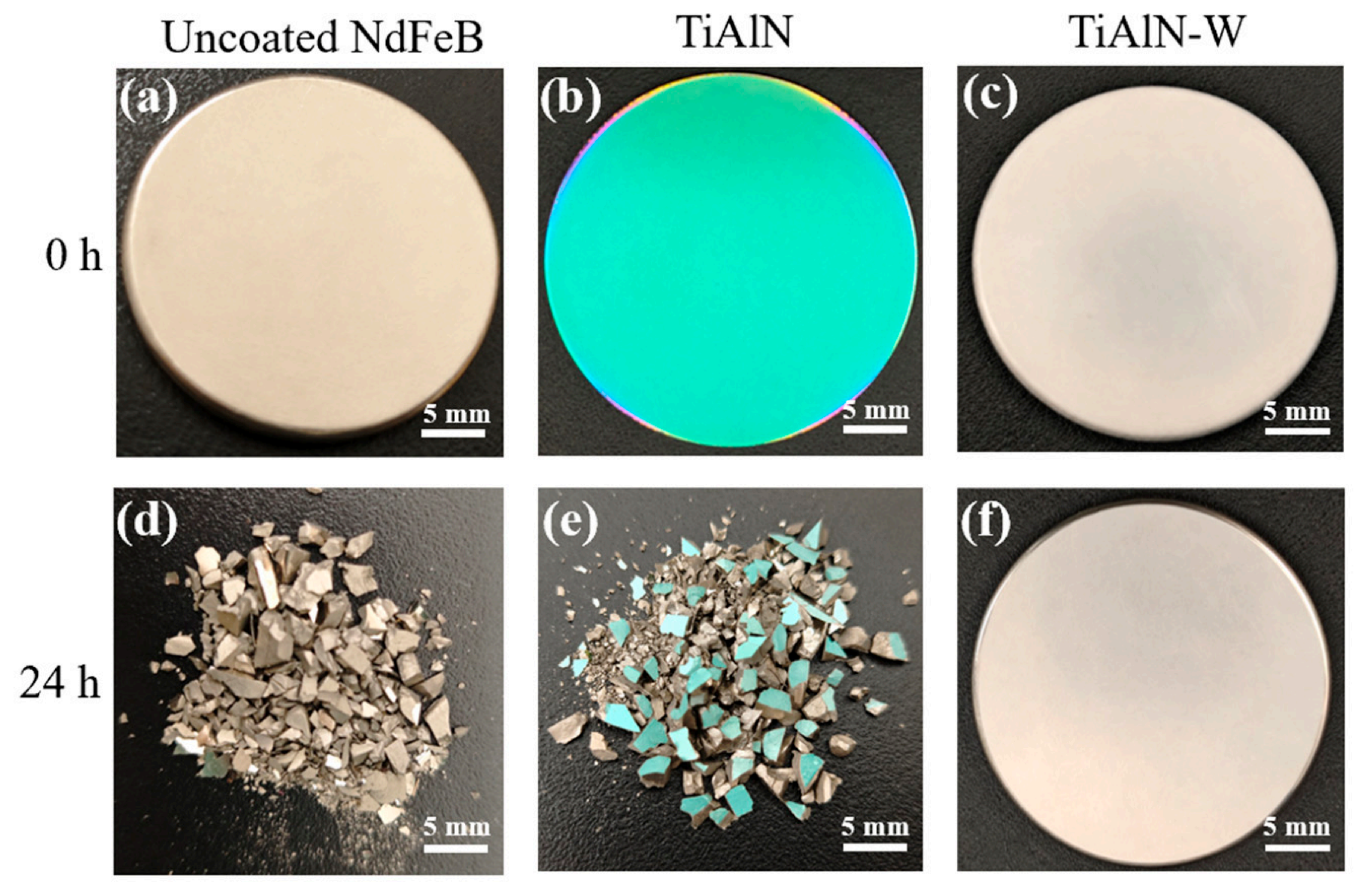
Optical photographs of (**a**,**d**) uncoated NdFeB, (**b**,**e**) TiAlN-coated NdFeB and (**c**,**f**) TiAlN-W-coated NdFeB samples before and after exposure to 5 MPa H_2_ at 23 °C for 24 h. The coated samples were fully covered by the corresponding coatings before exposure. The photographs were taken under the same lighting and environmental conditions. The visual damage score was defined as 0 = macroscopically intact, 1 = slight surface cracking, 2 = partial fracture and 3 = severe pulverization or loss of bulk integrity. This test was used as a qualitative assessment of hydrogen-induced pulverization resistance rather than a quantitative hydrogen permeation measurement.

As shown in Figure 8, all samples retained their original disk shape before hydrogen exposure. After exposure to 5 MPa H_2_ at 23 °C for 24 h, the uncoated NdFeB sample was severely pulverized. The TiAlN-coated NdFeB sample also lost its bulk integrity and fractured into small fragments. In contrast, the TiAlN-W-coated NdFeB sample remained macroscopically intact after the same exposure. This result indicates that TiAlN-W provides better resistance to hydrogen-induced pulverization of NdFeB than TiAlN.

To provide a semi-quantitative comparison of macroscopic damage, a visual damage score was introduced. The score was defined as follows: 0 = macroscopically intact, 1 = slight surface cracking, 2 = partial fracture and 3 = severe pulverization or loss of bulk integrity. According to this criterion, the uncoated NdFeB and TiAlN-coated NdFeB samples were assigned a damage score of 3 after hydrogen exposure, whereas the TiAlN-W-coated NdFeB sample was assigned a damage score of 0. This comparison further supports the improved macroscopic damage resistance of the TiAlN-W coating under the present exposure condition.

The observation is consistent with the electrochemical hydrogen permeation results on Fe substrates, where TiAlN-W showed a much lower permeation current than TiAlN. However, it should be noted that this gas-phase exposure test was used as an application-oriented validation. The hydrogen uptake in NdFeB was not monitored in real time, and the result is therefore discussed in terms of macroscopic damage resistance rather than quantitative hydrogen permeability.

Mass change was not used as a reliable parameter in this work because the uncoated and TiAlN-coated samples were pulverized after hydrogen exposure, and post-test weighing could be affected by fragment loss during handling. Magnetic property retention was also not evaluated because the NdFeB magnets were demagnetized before coating deposition to reduce magnetic-field interference during sputtering. Therefore, the present high-pressure H_2_ exposure test should be regarded as an application-oriented qualitative/semi-quantitative validation rather than a full long-term service evaluation.

The present test was conducted at a single condition of 5 MPa H_2_, 23 °C and 24 h. Practical hydrogen service environments may involve longer exposure times, pressure cycling, temperature variation, humidity and mechanical loading. Further systematic tests under varied hydrogen pressures, temperatures and exposure durations are needed to evaluate the long-term stability and practical applicability of TiAlN-W coatings under realistic hydrogen-related service conditions.

In this work, magnetic property measurements were not performed because the NdFeB magnets were intentionally demagnetized before coating deposition to reduce magnetic interference during PVD processing. The demagnetization treatment was conducted at 180 °C for 20 min in vacuum, which is below the Curie temperature of the Nd_2_Fe_14_B main phase and is not expected to induce a phase transformation under the present conditions. Since TiAlN and TiAlN-W are non-ferromagnetic ceramic coatings with micrometer-scale thicknesses, their direct contribution to the total magnetic moment of bulk NdFeB is expected to be limited. Nevertheless, systematic VSM or B–H loop measurements after coating deposition, hydrogen exposure and remagnetization are required to quantitatively evaluate magnetic performance, and this will be addressed in future work.

### 3.6. Structure–Permeation Relationship

Hydrogen permeation through a coated metal is influenced not only by the intrinsic hydrogen transport properties of the coating but also by its defect structure. Pinholes, cracks, connected grain boundaries and open columnar defects can provide preferential transport pathways in thin-film barrier coatings [32,33,34]. Therefore, coating compactness and the continuity of through-thickness defects are important factors controlling hydrogen permeation. Although W-induced microstructural modification of Ti–Al–N-based coatings has been reported previously for hard protective applications [24,25], its relevance to hydrogen permeation resistance, particularly for NdFeB protection, remains insufficiently clarified.

The TiAlN coating exhibited a columnar cross-sectional morphology and a measurable hydrogen permeation current. The intercolumnar boundaries extending along the growth direction may provide preferential pathways for hydrogen transport, which is consistent with the delayed but clearly detectable breakthrough observed for TiAlN. In comparison, TiAlN-W exhibited a more compact cross-sectional morphology at the SEM resolution, with fewer obvious continuous columnar features. Its broad GIXRD response further suggests a low-crystallinity W-modified Ti–Al–N structure with amorphous/nanocrystalline features. These observations are consistent with increased transport tortuosity and reduced continuity of through-thickness pathways, although nanoscale grain boundaries and porosity cannot be directly resolved by the present SEM and GIXRD measurements.

Although TiAlN-W showed a coarser surface morphology than TiAlN, surface roughness alone is unlikely to account for its substantially lower permeation current. A rougher surface may increase the real surface area and the number of electrochemically active sites, whereas TiAlN-W exhibited a near-background permeation current. Therefore, the observed improvement is more consistently correlated with the compact cross-sectional morphology than with surface roughness.

XPS shows that W modification changes the surface and near-surface chemical environment of the coating. Low-valence W-related species, such as W–N/W^n+^-related bonding, together with Ti 3p/W–O_x_-related contributions and oxide/oxynitride species, were detected. However, the Ti 3p/W–O_x_ overlap, possible sputter-induced reduction and the surface-sensitive nature of XPS prevent a direct relationship from being established between these chemical states and hydrogen solubility, trapping or diffusion. Previous first-principles studies indicate that hydrogen behavior in TiAlN and W-containing systems can be sensitive to local bonding, defects and near-surface states [21,35]. Nevertheless, these studies provide only indirect support for the present coating system, and the contribution of W-related surface chemistry to the measured permeation behavior cannot be established from the present XPS results alone.

The quantitative permeation results nevertheless demonstrate a clear difference between the two coatings. Under identical testing conditions, TiAlN-W reduced the permeation current density from 0.55 ± 0.08 μA cm^−2^ for TiAlN to below 0.01 μA cm^−2^, corresponding to a reduction of more than 55 times. Because both coatings had comparable thicknesses of 1.5 ± 0.2 μm, this difference cannot be mainly attributed to coating thickness. Instead, it is consistent with the observed differences in coating growth morphology and structural compactness.

Accordingly, the present results establish a qualitative correlation among W modification, coating structure and hydrogen permeation resistance rather than a fully resolved microscopic transport mechanism. The exact grain-boundary structure, nanoscale porosity, crystallite-size distribution, amorphous fraction and local coating density cannot be quantified from the present SEM, GIXRD and XPS results. Residual stress may also influence defect opening and hydrogen transport, but it was not measured in this work. Further cross-sectional HRTEM/STEM and SAED analyses before and after hydrogen exposure, together with nanoscale porosity and density measurements, residual-stress characterization and dedicated hydrogen-transport calculations, will be required to clarify the detailed transport mechanism.

### 3.7. Practical Implications and Limitations

The practical implications of the TiAlN-W coating should also be considered. The coating was prepared by PVD, a mature and scalable technique for nitride coatings, and its thickness was only approximately 1.5 μm, which is favorable for maintaining the dimensional accuracy of NdFeB components. The NdFeB substrates were demagnetized before deposition to reduce magnetic-field interference during sputtering, indicating that the deposition process can be adapted to magnetic substrates.

However, several issues remain before practical implementation. W incorporation may increase target cost and process complexity compared with conventional TiAlN coatings. For commercial NdFeB components with complex geometries, full-surface coverage, edge protection, batch uniformity, coating adhesion and residual stress require further optimization. The influence of deposition and subsequent hydrogen exposure on magnetic properties should also be evaluated systematically by VSM or B–H loop measurements after remagnetization.

The present 5 MPa H_2_ exposure test at 23 °C for 24 h should therefore be regarded as an application-oriented qualitative/semi-quantitative validation rather than a long-term service assessment. The TiAlN-W-coated NdFeB sample remained macroscopically intact under this condition, whereas the uncoated and TiAlN-coated samples were severely damaged. However, this result demonstrates suppression or delay of hydrogen-induced pulverization rather than complete prevention of hydrogen ingress. Future work should include longer-duration and cyclic hydrogen exposure under different pressures and temperatures, together with post-exposure SEM/TEM, hydrogen-content analysis, mass retention, adhesion and magnetic-property measurements.

Overall, the present results demonstrate the potential of W-modified TiAlN as a hydrogen permeation barrier coating for NdFeB magnets, while further nanoscale structural characterization and long-term service evaluation are required before industrial qualification.

## 4. Conclusions

TiAlN and TiAlN-W coatings with comparable thicknesses were prepared by PVD, and their hydrogen barrier behavior was evaluated using Fe and NdFeB substrates. The main conclusions are as follows:

(1) W incorporation changed TiAlN from a weakly crystalline columnar coating to a compact low-crystallinity W-modified Ti–Al–N coating with suppressed columnar boundaries.
(2) XPS results indicated the presence of low-valence W-related species, such as W–N/Wδ^+^ bonding, together with Ti 3p/W–O_x_-related contributions and surface oxide/oxynitride species. This suggests that W incorporation modifies the surface and near-surface chemical environment of TiAlN.
(3) Electrochemical hydrogen permeation tests showed that TiAlN reduced the steady-state current density from 2.54±0.86 to $0.55\pm 0.08\;\mu Acm^{-2}$, with a PRF of 4.68±0.16. TiAlN-W showed no obvious hydrogen breakthrough, with a current density below $0.01\;\mu Acm^{-2}$ and a lower-bound PRF of >250.
(4) After exposure to 5 MPa H_2_ at 23 °C for 24 h, the uncoated and TiAlN-coated NdFeB samples were pulverized, whereas the TiAlN-W-coated sample remained macroscopically intact.
(5) The enhanced hydrogen barrier performance of TiAlN-W is attributed to its compact non-columnar structure, suppressed continuous diffusion channels, and W-modified near-surface chemistry. This coating shows promise for protecting magnetic materials in hydrogen-containing environments.

## Figures and Tables

**Table 1:** Deposition parameters of TiAlN and TiAlN-W thin films.

Coatings	Target Configuration/Purity	Source	Base Vacuum (Pa)	Bias Voltage (V)	Working Pressure (Pa)	Ar/N_2_ (sccm)	Power (W)	Time (min)
TiAlN	Separate Ti target (99.99%), Separate Al target (99.99%)	RF	3.0 × 10^−3^	100	0.8	30/6	Ti: 150, Al: 120,	180
TiAlN-W	Separate Ti target (99.999%), Separate Al target (99.99%), Separate W target (99.99%)	RF	3.0 × 10^−3^	100	0.8	30/6	Ti: 150, Al: 120, W: 60,	40

Note: The coatings were deposited by co-sputtering from separate elemental targets. The same Ar/N_2_ flow ratio was used for both coatings. RF stands for radio frequency power supply.

**Table 2:** Surface elemental compositions of TiAlN and TiAlN-W coatings determined by EDS.

EI	Ti	Al	N	W
Series	K	K	K	K
TiAlN	30.87 ± 0.11	22.53 ± 0.09	47.60 ± 0.13	-
TiAlN-W	20.47 ± 0.11	19.87 ± 0.11	44.31 ± 0.11	15.47 ± 0.06

**Table 3:** Electrochemical hydrogen permeation parameters of the bare Fe substrate, TiAlN-coated Fe and TiAlN-W-coated Fe samples. Values are presented as the mean ± standard deviation from three independent measurements. The practical lower detection limit of the permeation current density was taken as $0.01\;\mu Acm^{-2}$, based on the stabilized background/noise level of the detection cell. For TiAlN-W, the current density remained below this limit; therefore, the steady-state current density is reported as $<0.01\;\mu Acm^{-2}$, and the PRF is given as a conservative lower-bound value. PRF uncertainty for measurable currents was calculated by error propagation.

Sample	Steady-State Current Density i (μA/cm^2^)	Breakthrough/lag Time $t_{b}$ (s)	PRF
Substrate	2.54 ± 0.86	270 ± 5	1
TiAlN	0.55 ± 0.08	1431 ± 8	4.6 ± 1.7
TiAlN-W	<0.01	not detected within test time	>250

Note: The breakthrough time is defined as the time interval between switching on the hydrogen charging current and the first obvious increase in hydrogen permeation current, corresponding to the initial rising point of the permeation curve. PRF, permeation reduction factor.

**Table 4:** Comparison of hydrogen barrier performance of representative coatings reported in the literature and this work.

Coating System	Preparation/Structure	Thickness	Test Temperature	Reported PRF	Main Feature	Ref.
TiN	PVD monolayer	1.5–5.5 μm	20 °C	2.11–10.30	Performance strongly affected by pores, defects and thickness	[28]
TiAlN/AlTiN	Dense nitride coating	~5 μm	400 °C	5800–20,000	Dense Al-containing nitride with high intrinsic barrier potential	[29]
CrN/AlTiN multilayer	Multilayer nitride	—	—	~10–100 times higher than corresponding monolayers	Interrupted diffusion paths and multilayer interfaces	[30]
Amorphous SiC	Amorphous coating	~1.5 μm	723–823 K	~10^3^	High barrier efficiency; possible degradation at higher temperature due to cracking	[31]
TiAlN	PVD monolayer	1.5 ± 0.2 μm	23 °C	4.68 ± 0.16	Columnar TiAlN with measurable permeation	This work
TiAlN-W	PVD W-modified TiAlN	1.5 ± 0.2 μm	23 °C	>250	Compact W-modified Ti–Al–N with suppressed continuous diffusion paths	This work

Note: PRF (permeation reduction factor) values are affected by coating thickness, substrate material, test temperature, hydrogen source, testing method and defect density. Therefore, the comparison is semi-quantitative and should not be treated as an absolute ranking.

## Data Availability

The original contributions presented in this study are included in the article/Supplementary Material. Further inquiries can be directed to the corresponding author.

## Supplementary Materials

The following supporting information can be downloaded at: https://www.mdpi.com/article/10.3390/ma19153265/s1, Table S1: XPS peak-fitting parameters of TiAlN and TiAlN-W coatings. Figure S1: Full electrochemical hydrogen permeation curves from three independent tests for bare Fe, TiAlN-coated Fe and TiAlN-W-coated Fe specimens.
