# Supplementary material for: W-Modified TiAlN Coatings with Compact Non-Columnar Structure for Enhanced Hydrogen Barrier Protection of NdFeB Magnets
Source: Materials (Basel). 2026 Aug 2;19(15):3265. doi: 10.3390/ma19153265 (PMC13466834; doi:10.3390/ma19153265)
Supplement: Supplementary file 1 [file materials-19-03265-s001.zip › materials-4421781-supplementary.pdf]

Supplementary Note:

Multiple EDS point/area measurements were performed on the cross-sections of the 150 W and 300 W films to evaluate the compositional dispersion within the analyzed film regions. All measured regions contain Fe, Cu, V, Ti and Nb, confirming that the sputtered films retain a multi-principal-element composition. The 300 W film shows relatively higher V and Nb contents and lower Ti and Cu contents than the 150 W film. However, because SEM-EDS is semi-quantitative and no depth profiling was performed, these data are used only for evaluating relative composition variations rather than determining definitive absolute film compositions or through-thickness homogeneity.

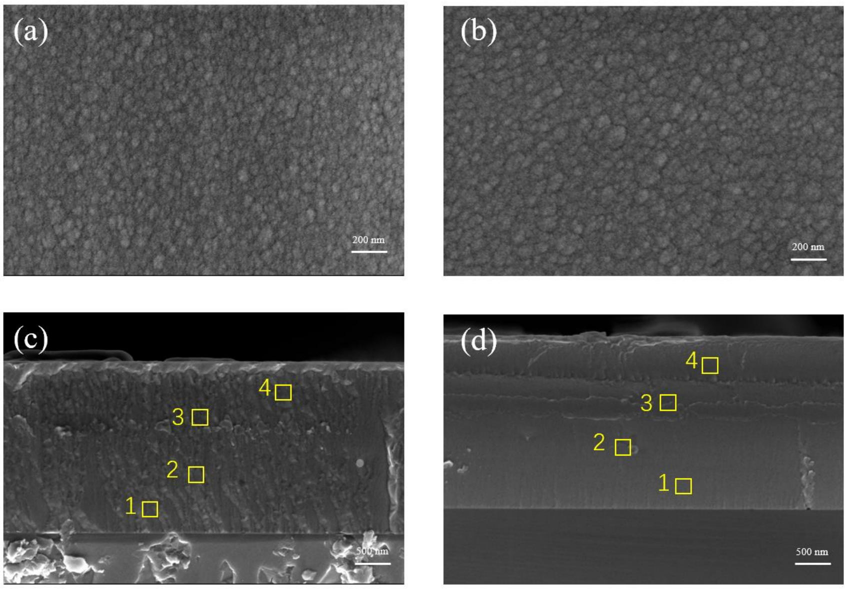

Figure S1. Surface and cross-sectional SEM images of $Fe_5Cu_5V_{30}Ti_{30}Nb_{30}$ high-entropy alloy films deposited at different sputtering powers: (a, c) 150 W and (b, d) 300 W. Panels (a) and (b) show the surface morphologies, while panels (c) and (d) show the cross-sectional morphologies and the positions selected for EDS point/area analysis. The numbered markers in (c) and (d) correspond to the EDS analysis areas listed in Table S1.

Table S1. Semi-quantitative cross-sectional EDS composition estimates of $Fe_5Cu_5V_{30}Ti_{30}Nb_{30}$ films deposited at different sputtering powers based on multiple point/area measurements. The analyzed positions are marked in Figure S1c, d. The data are presented in atomic percent (at. %). These values are used to evaluate the compositional dispersion within the analyzed cross-sectional regions and should not be interpreted as definitive absolute film compositions or complete depth-profile measurements.

EDS position	Fe (at. %)	Cu (at. %)	V (at. %)	Ti (at. %)	Nb (at. %)
**150 W-1**	5.2	5..6	32.7	27.8	28.7
**150 W-2**	5.0	5.6	32.9	27.7	28.8
**150 W-3**	4.9	5.8	32.7	28.1	28.9

**150 W-4**	5.1	5.7	32.6	27.9	28.7
**300 W-1**	5.7	4.3	35.8	23.4	30.8
**300 W-2**	5.8	4.2	35.6	23.3	30.5
**300 W-3**	5.5	4.3	36.0	23.6	30.6
**300 W-4**	5.6	4.5	36.1	23.4	30.7
